# Establishing a Macrophage Phenotypic Switch-Associated Signature-Based Risk Model for Predicting the Prognoses of Lung Adenocarcinoma

**DOI:** 10.3389/fonc.2021.771988

**Published:** 2022-02-23

**Authors:** Jun Chen, Chao Zhou, Ying Liu

**Affiliations:** ^1^ Department of Oncology, The First Affiliated Hospital of Nanchang University, Nanchang, China; ^2^ Department of Neurology, Jiangxi Provincial People’s Hospital Affiliated to Nanchang University, Nanchang, China; ^3^ Department of Emergency, The First Affiliated Hospital of Nanchang University, Nanchang, China

**Keywords:** lung adenocarcinoma, tumor-associated macrophages, macrophage phenotypic switch, macrophage phenotypic switch-related gene, MRG signature

## Abstract

**Background:**

Tumor-associated macrophages are important components of the tumor microenvironment, and the macrophage phenotypic switch has been shown to correlate with tumor development. However, the use of a macrophage phenotypic switch-related gene (MRG)-based prognosis signature for lung adenocarcinoma (LADC) has not yet been investigated.

**Methods:**

In total, 1,114 LADC cases from two different databases were collected. The samples from TCGA were used as the training set (N = 490), whereas two independent datasets (GSE31210 and GSE72094) from the GEO database were used as the validation sets (N = 624). A robust MRG signature that predicted clinical outcomes of LADC patients was identified through multivariate COX and Lasso regression analysis. Gene set enrichment analysis was applied to analyze molecular pathways associated with the MRG signature. Moreover, the fractions of 22 immune cells were estimated using CIBERSORT algorithm.

**Results:**

An eight MRG-based signature comprising CTSL, ECT2, HCFC2, HNRNPK, LRIG1, OSBPL5, P4HA1, and TUBA4A was used to estimate the LADC patients’ overall survival. The MRG model was capable of distinguishing high-risk patients from low-risk patients and accurately predict survival in both the training and validation cohorts. Subsequently, the eight MRG-based signature and other features were used to construct a nomogram to better predict the survival of LADC patients. Calibration plots and decision curve analysis exhibited good consistency between the nomogram predictions and actual observation. ROC curves displayed that the signature had good robustness to predict LADC patients’ prognostic outcome.

**Conclusions:**

We identified a phenotypic switch-related signature for predicting the survival of patients with LADC.

## Introduction

Lung cancer is the most prevalent cancers with the highest mortality worldwide (1). Non-small cell lung cancer (NSCLC) accounts for nearly 90% of lung cancer cases and is divided into three main types, lung adenocarcinoma (LADC), lung squamous cell carcinoma, and large cell carcinoma (2). As the most prevailing histological type of NSCLC, LADC comprises up to 40–50% of all lung cancer cases (3, 4). Despite the tremendous effort aimed at discovering predictors of recurrence risk that allow prompt therapeutic intervention, most patients are diagnosed with advanced-stage diseases and different types of distant organ metastases (5); thus, the overall 5-year survival rate of LADC remains at approximately 15% (6). This might be primarily due to the high heterogeneity of LADC and the advanced disease stage at which the patients are diagnosed (7).

Advancements in high-throughput sequencing technologies present novel therapeutic strategies for lung cancer (8). Thus, mining genes with LADC prognostic value is necessary to better help improve risk-stratification of patients based on the clinical outcome and develop novel therapeutic targets.

The tumor microenvironment (TME) represents the extra-cellular environment in which tumor cells reside and it comprises tumor cells, immune cells, extracellular matrix, and growth factors (9). TME plays a crucial role in the progression and migration of LADC (10). Macrophages within the TME, termed tumor-associated macrophages (TAMs), are an important component of the TME (11). TAMs can be polarized to M1/M2 phenotypes based on their functional status as induced by the microenvironment (12). M1 macrophages, highly expressed major histocompatibility complex class II, CD68 labeling and CD80/CD86 costimulatory molecules, located within tumors are thought to induce tumor suppression by activating anti-tumor immunity (13). However, most TAMs in the TME manifest an M2-like phenotype (characterized by up-regulated expression of CD200R membrane glycoprotein, Arg-1, YM1, Fizz1 and other receptors) that facilitates immunological tolerance and promotes tumor progression (14). Tumor cells recruit macrophages by releasing various chemokines, cytokines, and growth factors, and they develop them into pro-tumorigenic M2 macrophages. Therefore, the macrophage phenotypic switch is correlated with tumor development, whereas macrophage phenotypic switch-related genes (MRGs) might provide insightful information to estimate LADC patients’ prognosis.

Herein, we analyzed the MRG expression alterations obtained from The Cancer Genome Atlas (TCGA) and Gene Expression Omnibus (GEO) databases regarding LADC patients and identified dysregulated MRGs with prognostic value. Furthermore, we developed a novel and robust gene prognostic signature based on the identified dysregulated MRGs. Finally, a prognostic nomogram integrating the signature and multiple clinical parameters meant to estimate the overall survival (OS) of LADC patients was developed. These results might be meaningful for the development of comprehensive therapeutic approaches for LADC patients.

## Methods

### Data Collection

The transcriptome profiles and corresponding clinical data of LADC patients were downloaded from TCGA (https://portal.gdc.cancer.gov/) and GEO (https://www.ncbi.nlm.nih.gov/geo/) databases. TCGA-LADC comprised a total of 594 (535 tumor sample and 59 normal samples) adenocarcinoma cases. The main characteristics of the analysis included the following: age, sex, and pathologic stage; details of patient clinical information are described in **Table 1**. GSE31210 comprised a total of 226 primary LADC of pathological stage I-II. The median age was 67 years and the range was 30-76 years, and there were 105 male and 121 female patients. GSE72094 comprised a total of 442 LADC cases. The median age was 70 years and the range was 38-89 years, and there were 202 male and 240 female patients. The samples from TCGA database were defined as the training set, the samples from the GSE31210 database were defined as the validation set, whereas LADC cases from GSE72094 were set as testing set. LADC patients with missing survival values or follow-up time < 1 days were excluded. A total of 1,114 samples (490 from TCGA, 226 from GSE31210, and 398 from GSE72094) were used in our study.

**Table 1:** Prognostic roles of the MRGs signature with different demographic and clinical characteristics in TCGA training set. .

Characteristics	No.		%	HR (95% CI)	*P*-value
	high-risk	low-risk			
**Age (years)**					
< 65	114	105	44.69%	0.549 (0.334-0.901)	0.018
≥ 65	131	140	55.31%	0.359 (0.233-0.552)	0.000
**Sex**					
Male	127	97	45.71%	0252 (0.148-0.429)	0.000
Female	118	148	54.29%	0.699 (0.455-1.076)	0.104
**Stage**					
I	106	155	53.27%	0.517 (0.302-0.886)	0.016
II	69	48	23.88%	0.592 (0.321-1.091)	0.093
III	54	25	16.12%	0.510 (0.244-1.067)	0.074
IV	15	10	5.10%	0.323 (0.089-1.163)	0.084
NA	1	7	1.63%	−	−
**T stage**					
T1	62	104	33.88%	0.780 (0.414-1.472)	0.444
T2	144	114	52.65%	0.389 (0.247-0.613)	0.000
T3	29	16	9.18%	0.077 (0.010-0.590)	0.014
T4	9	9	3.67%	0.574 (0.141-2.343)	0.440
NA	1	2	0.61%	−	−
**M stage**					
M0	171	151	65.71%	0.494 (0.331-0.738)	0.001
M1	15	9	4.90%	0.233 (0.051-1.056)	0.059
NA	59	85	29.39%	−	−
**N stage**					
N0	133	184	64.69%	0.475 (0.299-0.756)	0.002
N1	59	33	18.78%	0.609 (0.328-1.132)	0.117
N2	48	20	13.88%	0.542 (0.246-1.193)	0.128
N3	1	1	0.041%	−	−
NA	4	7	2.24%	−	−

NA, Not available.

The protein expression data of the MRGs of LADC patients were evaluated using the Human Protein Atlas (https://www.proteinatlas.org/), which is derived from antibody-based protein profiling using immunohistochemistry.

### Acquisition of MRGs

MRGs were obtained from two MRG datasets (188 from GSE5099_CLASSICAL_M1_VS_ALTERNATIVE_M2_MACROPHAGE_UP and 194 from GSE5099_CLASSICAL_M1_VS_ALTERNATIVE_M2_MACROPHAGE_DN) (15) from the gene set enrichment analysis (GSEA) website (http://www.gsea-msigdb.org/gsea/msigdb/). Finally, a total of 382 MRGs were utilized in this study (**Supplementary Table 1**).

### Development and Validation of a Prognostic Model

The univariate Cox regression analysis was used to screen out the genes significantly correlated with OS based on the 382 MRGs (for P-values < 0.05). Next, the overlapped prognosis-related MRGs from TCGA and GEO databases were selected for the least absolute shrinkage and selection operator (Lasso) with ten-fold cross-validation which was subsequently applied using “glmnet” and “survival” packages. Afterwards, a multivariate Cox regression was applied out to select candidate OS-related MRGs and determine a prognostic signature. The risk score was calculated as follows: Risk score = β1 × (expression of RNA1) + β2 × (expression of RNA2) + ··· + βn × (expression of RNAn). Median MRG risk scores were used to differentiate high-risk subgroups from LADC patients. The regression coefficient (β) was obtained from the multivariate Cox regression analysis. Additionally, Kaplan-Meier survival analysis was conducted to assess the predictive performance of the prognostic signature. The signature was also externally validated with the GEO dataset using the same formula. All analyses were carried out using R language, version 4.0.5 (www.r-project.org).

### GSEA

Gene set enrichment analysis (GSEA) was conducted to investigate various molecular pathways differentially activated between high- and low-risk subgroups. False discovery rate q-values < 0.05 and |NES| > 1 were defined as statistically significant difference.

### Estimating the Proportion of Immune Cells

We utilized CIBERSORT algorithm to estimate the proportion of 22 immune cells between low- and high-risk patients. The sum of ratio of 22 immune cell types in each sample is 1.

### Construction and Evaluation of a Nomogram

To provide a more individualized predictive model, a nomogram combining the MRG signature and other clinical variables was constructed using the training cohort. The discrimination ability of the nomogram was assessed using the calibration curves and receiver operating characteristic (ROC) curves in the training and validation subgroups. Next, decision curve analysis (DCA) was applied to evaluate the clinical usefulness of the nomogram in the training and testing sets.

### Statistical Analysis

Continuous variables were presented as means ± SD, whereas categorical variables were displayed as percentages. The statistical significance of the differences in survival rate was measured using the log-rank test with a threshold of P-value < 0.05. Kaplan-Meier plots were applied to display the differences in survival duration. All statistical analyses were conducted using the software R (version 3.5.2) with corresponding packages.

## Results

### Establishment of an MRG-Based Prognosis Signature

**Figure 1:**
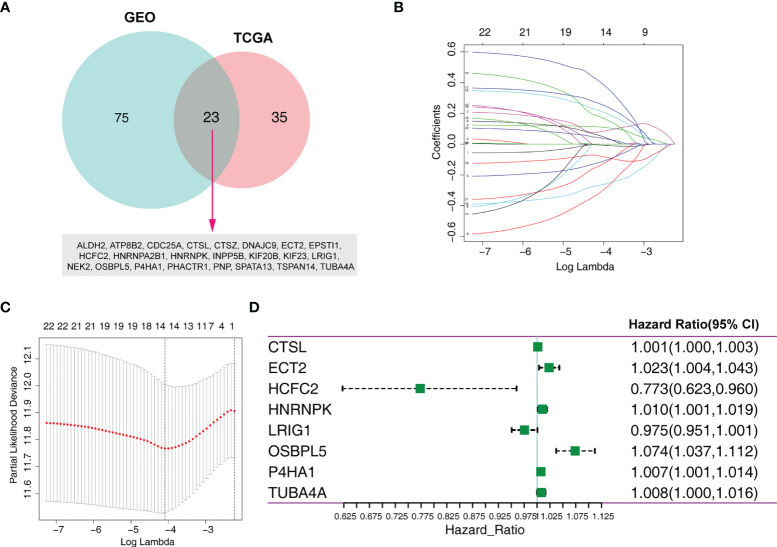
Identification of an MRG-based prognosis signature. **(A)** Twenty-three overlapping overall survival-related MRGs from TCGA and GEO databases were obtained following univariate Cox regression analysis. **(B)** Lasso coefficient profiles of the 23 prognosis-associated MRGs from the training set. **(C)** Partial likelihood deviance of variables revealed by the Lasso regression model. **(D)** Forest plot of multivariate Cox regression analysis.

To limit the candidate prognosis-related MRGs, the OS-related MRGs that were overlapping in the data from TCGA and GEO databases (23 MRGs) were identified (**Figure 1A**). Next, the 23 MRGs were used to the Lasso-Cox proportional hazards regression and ten-fold cross-validation to construct the best gene signature, and 14 candidate MRGs were ultimately identified (**Figures 1B, C**). Further, a multivariate Cox regression was used, and results exhibited that CTSL, ECT2, HCFC2, HNRNPK, LRIG1, OSBPL5, P4HA1, and TUBA4A were the independent prognostic MRGs (**Figure 1D**). We created a risk score according to the expression of the eight MRGs as follows: Risk score = CTSL × 0.001326639 + ECT2 × 0.023009173 - HCFC2 × 0.257179317 + HNRNPK × 0.010298027 - LRIG1 × 0.024832171 + OSBPL5 × 0.071303241 + P4HA1 × 0.007389189 + TUBA4A × 0.008003706.

### MRG Expression

**Figure 2:**
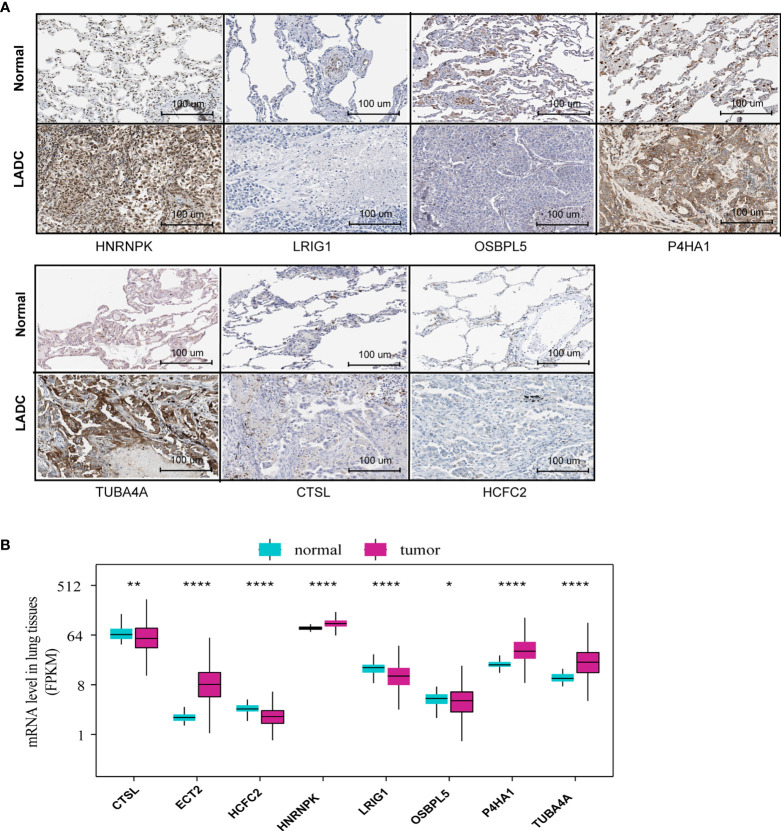
MRG protein and mRNA level between normal and tumor tissues. **(A)** The MRG protein levels detected *via* immunohistochemistry provided by the HPA database (ETC2 was unavailable in HPA). **(B)** MRG mRNA levels of normal and tumor tissues based on RNA-Seq data from the training set *P<0.05, **P<0.01, ***P<0.001, ****P<0.0001.

We investigated the protein levels of these genes, detected using immunohistochemistry and obtained from the HPA database. The immumohistochemical staining of MRGs were based on the normal alveolar and tumor tissues. We discovered that the protein levels of HNRNPK, P4HA1, and TUBA4A were significantly upregulated, while CTSL, HNRNPK, and OSBPL5 were significantly downregulated in the tumor tissues compared to those of normal tissues (**Figure 2A**). The quantitative analysis results for each immunohistochemistry were show in **Supplementary Material**. We also investigated the expression level of the identified MRGs for normal and tumor samples using RNA-Seq data from the training set. The results showed roughly the same trend as the one observed for the protein expression (**Figure 2B**).

### Prognostic Value of the 10-MRG Signature

**Figure 3:**
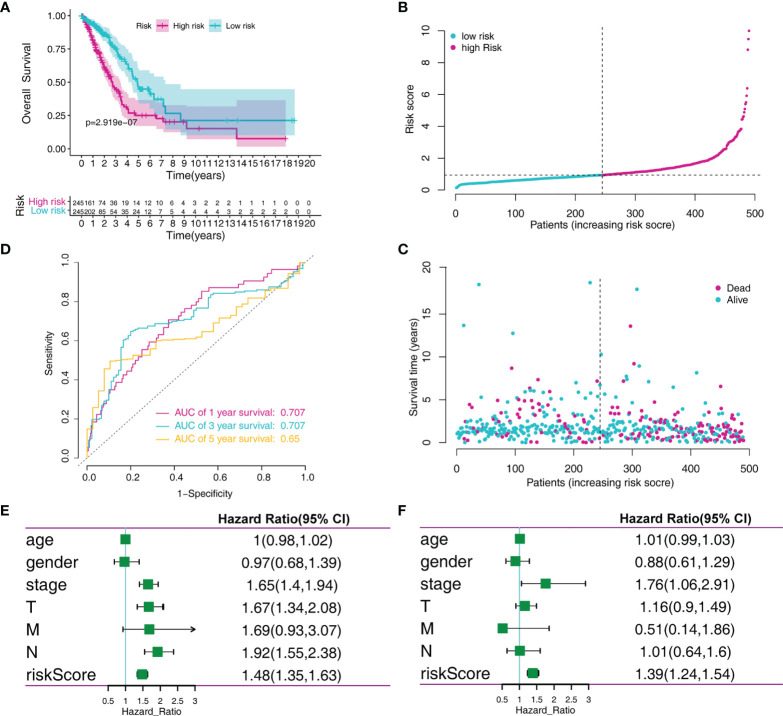
MRGs-based risk signature evaluation using the training set. **(A)** Kaplan-Meier survival analysis revealed the difference in survival rate between high- and low-risk patients. **(B)** Time-dependent ROC curve analysis for 1-, 3-, and 5-year predictions of overall survival using the MRG-based signature. **(C)** Risk score distribution of patients with the overall survival and signature. **(D)** Overall survival scatter plots for LADC patients. **(E)** Univariate Cox analyses of the MRG signature and clinical variables. **(F)** Multivariate Cox analyses of the MRG signature and clinical variables.

To identify the MRG signature suitable for LADC survival prediction, the LADC patients were separated into low-risk (N = 245) and high-risk groups (N = 245) based on the median risk score. Kaplan-Meier curve analysis depicted that high-risk patients were associated with poorer OS compared to the low-risk patients (P < 0.001, **Figure 3A**). Furthermore, the ROC curve analysis demonstrated that the area under the ROC curve (AUC) of the prognostic MRG model at 1, 3, and 5 years were 0.707, 0.707, and 0.65 in the training set, respectively (**Figure 3B**). The distribution survival status and time for each patient from the training set were plotted with a division line indicating the risk score cutoffs (**Figures 3C, D**). Next, we conducted the univariate and multivariate Cox regression analyses to analyze the signature and clinicopathological independent indices predicting survival. The results showed that the MRG-based signature was able to be an independent prognostic indicator (**Figures 3E, F**). Its prediction capacity was also evaluated through calculating C‐index in the training set. The results showed that the C-index for the prediction of OS of the identified MRG signature was 0.72 (95% CI = 0.65–0.76).

**Figure 4:**
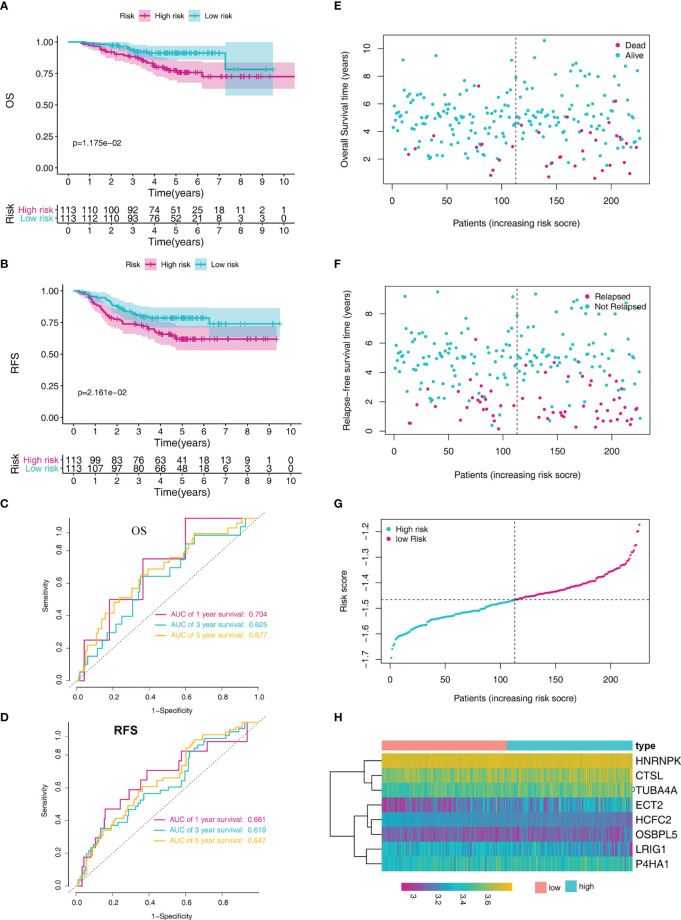
MRG-based risk signature evaluation using the validation set (GSE31210). **(A)** Kaplan-Meier analysis of the overall survival in LADC patients based on risk stratification. **(B)** Kaplan-Meier analysis for relapse-free survival of LADC patients based on risk stratification. **(C)** Time-dependent ROC curve analysis for 1-, 3-, and 5-year overall survival predictions obtained using the MRG-based signature. **(D)** Time-dependent ROC curve analysis for 1-, 3-, and 5-year relapse-free predictions obtained using the MRG-based signature. **(E)** Overall survival scatter plots for LADC patients. **(F)** Relapse-free survival scatter plots for LADC patients. **(G)** Risk score distribution of the LADC patients. **(H)** The heatmap of the eight MRGs.

We verified the prediction performance of this signature using LADC cases from the GSE31210 dataset. The risk score of each patient was calculated based on the indicated formula and separated into low-risk (N = 113) and high-risk groups (N=113) according to the median risk score. Kaplan-Meier analysis demonstrated that high-risk patients were associated with poorer OS and relapse-free survival (RFS) compared to the low-risk patients (**Figures 4A, B**). ROC curve analysis revealed that the AUC of the prognostic MRG model for predicting OS at 1, 3, and 5 years were 0.704, 0.625, and 0.677, respectively (**Figure 4C**). Furthermore, the ROC curve analysis revealed that the AUC of the prognostic MRG model for predicting RFS at 1, 3, and 5 years was 0.661, 0.619, and 0.647, respectively (**Figure 4D**). The survival status and time distribution for each patient from the validation set were plotted with a division line representing risk score cutoffs (**Figures 4E–G**). The expression profiles of the eight prognostic MRGs are illustrated in **Figure 4H**.

**Figure 5:**
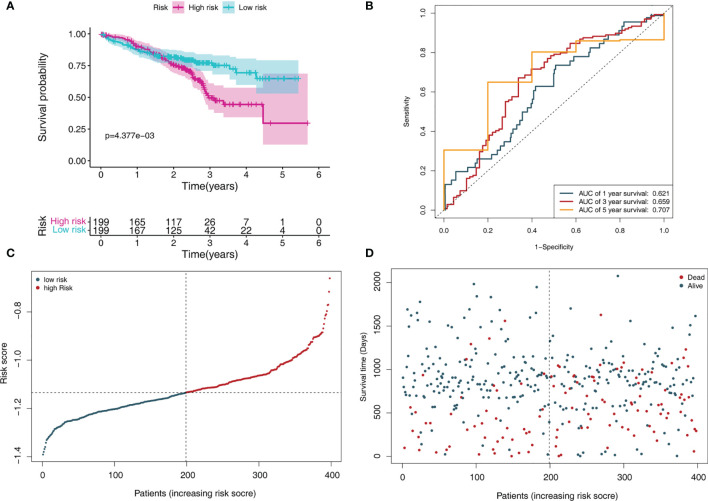
MRGs-based risk signature evaluation using the testing set (GSE72094). **(A)** Kaplan-Meier survival analysis revealed the difference in survival rate between high- and low-risk patients. **(B)** Time-dependent ROC curve analysis for 1-, 3-, and 5-year predictions of overall survival using the MRG-based signature. **(C)** Risk score distribution of patients with the overall survival and signature. **(D)** Overall survival scatter plots for LADC patients.

In addition, we further tested the prediction performance of this signature using LADC cases from the GSE72094 dataset. the LADC patients were separated into low-risk (N = 199) and high-risk groups (N = 199) based on the median risk score. Kaplan-Meier curve analysis depicted that high-risk patients were associated with poorer OS compared to the low-risk patients (P < 0.001, **Figure 5A**). Furthermore, the ROC curve analysis demonstrated that the area under the ROC curve (AUC) of the prognostic MRG model at 1, 3, and 5 years were 0.621, 0.659, and 0.707 in the test set, respectively (**Figure 5B**). The distribution survival status and time for each patient from the testing set were plotted with a division line indicating the risk score cutoffs (**Figures 5C, D**).

The prognostic significance of the signature was further assessed using subgroups with different demographics and clinical characteristics from the training set, including age, sex, TNM stage, and pathological stage (**Supplementary Figure 2**). We discovered that the MRG signature was useful for most subgroups (**Table 1**). For the validation set, the model can also accurately predict the OS and RFS of low- and high-risk groups in these subgroups (**Supplementary Table 2**).

### Correlation Between the MRGs and Clinicopathological Parameters

**Figure 6:**
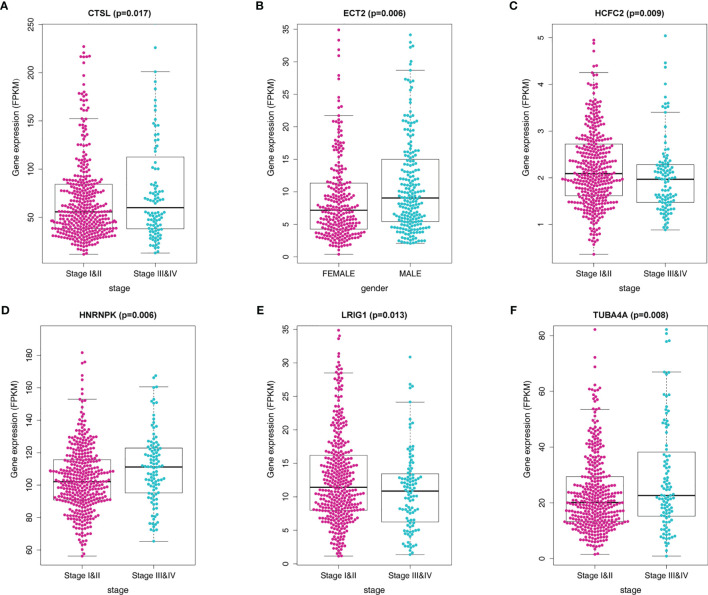
Correlation of the MRG mRNA expression levels with demographic and clinicopathological characteristics of LADC patients. **(A)** Correlation between CTSL mRNA levels and disease stage. **(B)** Correlation between ECT2 mRNA levels and gender. **(C)** Correlation between HCFC2 mRNA levels and disease stage. **(D)** Correlation between HNRNPK mRNA levels and disease stage. **(E)** Correlation between LRIG1 mRNA levels and disease stage **(F)** Correlation between TUBA4A mRNA levels and disease stage.

We further investigated the association between the MRGs and clinicopathological characteristics such as age, gender, pathological stage, and TNM stage for patients in the training cohort. We observed the differential expression of CTSL, ECT2, HCFC2, HNRNPK, LRIG1, and TUBA4A (**Figures 6A–F**).

### GSEA

**Figure 7:**
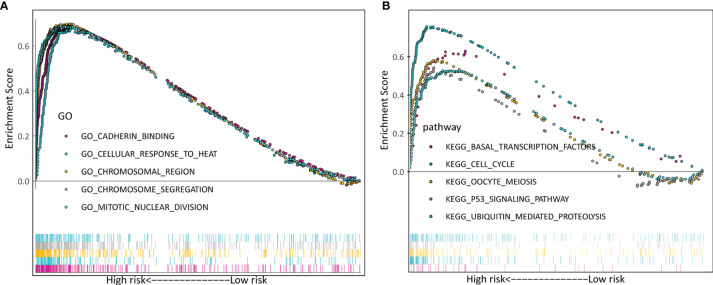
Verification of the biosignature stratified by different clinical parameters in the training set. **(A)** GO terms. **(B)** KEGG terms.

Additionally, we explored the differentially signaling pathways between high- and low-risk LADC patient through GSEA. In the high-risk group, the top five enriched GO terms included cadherin binding, cellular response to heat, chromosomal region, chromosome segregation, and mitotic nuclear division (**Figure 7A**). The top five enriched KEGG pathways were basal transcription factors, cell cycle, oocyte meiosis, p53 signaling pathway, and ubiquitin mediated proteolysis (**Figure 7B**).

### Immune Characteristics of Patients in the High- and Low-Risk Groups

**Figure 8:**
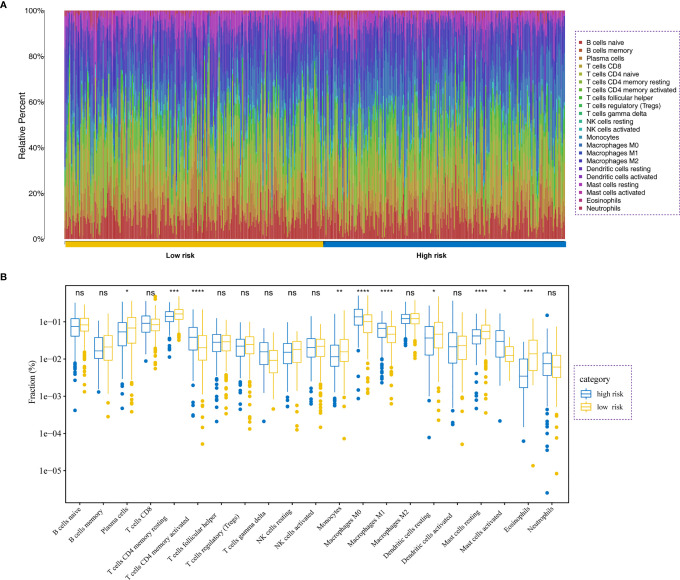
Immune analysis. **(A)** Relative proportion of immune cell infiltration in high- and low-risk patients. **(B)** Differences in immune cell infiltration between low- and high-risk LADC patients *P<0.05, **P<0.01, ***P<0.001, ****P<0.0001, ns, no significance.

We further investigate the tumor-infiltrating immune cells from the high- and low-risk patients using CIBERSORT. The results displayed that the tumors of high-risk patients exhibited a higher proportion of plasma cells, resting CD4 T memory cells, monocytes, resting dendritic cells, resting mast cells, and eosinophils; while activated CD4 T memory cells, M0 macrophages, M1 macrophages, and activated mast cells were higher in low-risk group (**Figures 8A, B**).

### Development and Validation of a Prognostic Nomogram Based on the Signature

**Figure 9:**
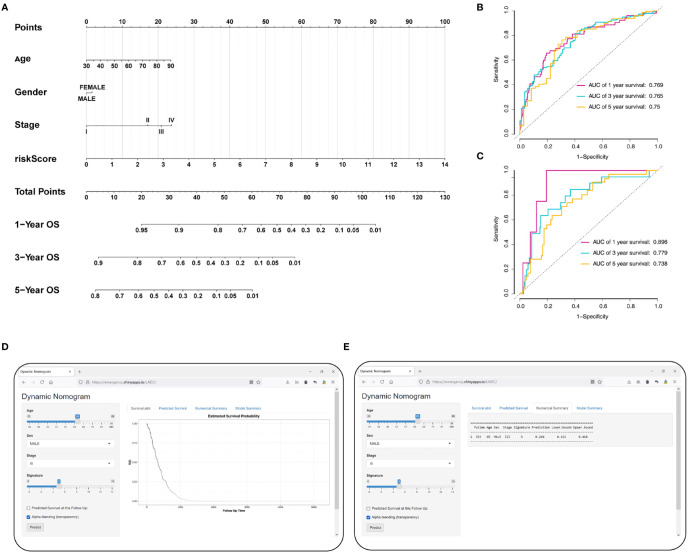
Construction of the MRG-based nomogram. **(A)** Development of MRG nomogram. **(B)** Time-dependent ROC curve analysis for 1-, 3-, and 5-year overall survival predictions obtained using the nomogram in the training set. **(C)** Time-dependent ROC curve analysis for 1-, 3-, and 5-year overall survival predictions obtained using the nomogram in the validation set. **(D)** Establishing an easy-to-operate web-based calculator for predicting the overall survival of LADC (https://emergency.shinyapps.io/LADC/). **(E)** 95% confidence interval of the web overall survival rate.

To accurately predict a certain clinical outcome, a nomogram was established by integrating stage, age, gender, and the eight MRGs using a Cox model (**Figure 9A**). For the training set, the AUCs of the nomogram at 1-, 3-, and 5-year OS were 0.769, 0.765, and 0.75, respectively (**Figure 9B**). In the validation set, the AUCs of the nomogram at 1-, 3-, and 5- year OS were 0.896, 0.779, and 0.738, respectively (**Figure 9C**). For convenient clinical application and visualization of the prognostic model, we established an easy-to-use web-based calculator (https://emergency.shinyapps.io/LADC/) for predicting the overall survival of LADC (**Figures 9D, E**).

We evaluate the predictive ability and clinical usefulness of the nomogram using calibration curves and DCA. The calibration plots displayed that the nomogram could accurately predict OS (**Supplementary Figures 3A, B**). In addition, we used DCA to assess the clinical usefulness of the nomogram; the results showed good clinical usefulness of this model both in the training and validation sets (**Supplementary Figures 3C, D**).

## Discussion

LADC is one of the most prevalent tumors with low survival rates in advanced stage patients (16). Accurately predicting LADC outcome will be helpful for more aggressive treatment, earlier intervention, and delayed tumor progression (17). The most commonly used tool to predict patient outcome is the AJCC staging system, which only focuses on clinical features; thus, making it difficult to develop individualized risk estimation. In the present study, we collected data on LADC patients and constructed a prognostic MRG-based signature and nomogram to better predict the OS of these patients. We showed that our MRG signature can predict the individual mortality risk of LADC patients and is helpful for devising individualized therapies against LADC.

Immune dysregulation is important in cancer progression. Most studies only focused on the T cell compartment (18, 19). However, macrophage phenotypic polarization represents a key step that accelerates tumor aggressiveness, which further imparts the MRGs satisfactory prognostic value (20). Here, we identified a signature composed of eight MRGs, CTSL, ECT2, HCFC2, HNRNPK, LRIG1, OSBPL5, P4HA1, and TUBA4A. Among these genes, the mRNA levels of ECT2, HNRNPK, P4HA1, and TUBA4A were significantly upregulated in the tumor tissues, when compared to those in normal tissues. However, the mRNA levels of CTSL, HNRNPK, LRIG1, and OSBPL5 were significantly downregulated in the tumor tissues compared to those observed in normal tissues. Furthermore, GSEA showed that tumor-associated pathways were enriched in samples from high-risk patients.

Cathepsin L (CTSL), one of the human cathepsin proteases, has been shown to be overexpressed in various carcinomas including ovary, cervix, breast, and colon tumors (21, 22). However, the function of CTSL in the complex process of tumorigenesis is not yet fully understood (23). It has been recently observed that CTSL is closely correlated with drug resistance in NSCLC (24). Our analyses showed that the upregulation of CTSL mRNA levels in LADC patients was associated with a higher risk of relapse and worse OS. Epithelial cell transforming sequence 2 (ECT2), a guanine nucleotide exchange factor of the Rho family of GTPases, has been shown to be involved in the oncogenic and malignant phenotypes of LADC (25). Furthermore, ETC2 has been reported to be amplified and its protein overexpressed in early invasive LADC (26). Previous studies indicated that ECT2 may promote the polarization of M2 macrophages by enhancing aerobic glycolysis and inhibiting the functions of immune cells in tumor (27). HNRNPK is a highly conserved RNA‐ and DNA‐binding protein (28) and its dysregulation has been shown to correlate with tumor development, progression, and prognosis (29–31). In line with these results, our analyses indicated an association between the HNRNPK mRNA levels and the high-risk score. Leucine-rich repeats and immunoglobulin-like domains 1 (LRIG1) is one of three members of a transmembrane protein family (32). LRIG1 is often regarded as a tumor suppressor in several tumors, including cervical cancer, melanoma, and cutaneous squamous cell carcinoma (33–35). However, we identified an association between the LRIG1 levels and high-risk scores. Oxysterol binding protein-like 5 (OSBPL5), a cytosolic mammalian protein, binds to an oxysterol ligand and interacts with the Golgi membrane; thus, playing a role in vesicle transport, lipid metabolism, and signal transduction (36). Nagano and colleagues reported that OSBPL5 are involved in the metastatic potential of lung cancer (37). P4HA1 was the most common subtype of prolyl 4-hydroxylase which enhanced collagen modification (38). Several studies reported that P4HA1 might serve as a pro-tumorigenic factor (39–41). However, studies investigating the roles of TUBA4A and HCFC2 and their functions in LADC are limited; thus, further studies are necessary to elucidate their associations with LADC.

The immune system can identify and eradicate tumor cells through innate and adaptive immune system. However, the TME could regulate this antitumor response by regulating the immune-infiltrating cells. Notably, TAMs and their progenitors account for the largest proportion of tumor-resident immune cells. M1 macrophages secrete inflammatory cytokines, including tumor necrosis factor-α as well as interleukin-12, and typically suppress tumor development. In the current study, we analyzed the differences in tumor-infiltrating immune cells between the high- and low-risk groups of patients. Our results showed that the high-risk group exhibited a higher proportion of plasma cells, resting CD4 T memory cells, monocytes, resting dendritic cells, resting mast cells, and eosinophils. Alternatively, the low-risk group showed higher proportions of activated CD4 T memory cells, M0 macrophages, M1 macrophages, and activated mast cells.

As far as we know, this study firstly analyzed the MRGs associated with the prognosis of LADC patients. More importantly, we developed an eight-gene signature to predict LADC patient outcomes with a satisfactory accuracy. However, some limitations of the present study are worth mentioning. First, all cases included in our study were retrospective samples, and the validation of our signature through prospective samples is still needed. Second, this risk score was calculated based on gene expression, without considering the mutations or epigenetic modifications that might represent key MRG drivers. Ultimately, a prognostic nomogram incorporating both the MRG signature and clinicopathological features for individual survival prediction was constructed and validated. The establishment of this model will help in better evaluation of the patients’ prognosis in the clinical setting and will aid in guiding follow-up and treatment processes.

## Data Availability Statement

Publicly available datasets were analyzed in this study. This data can be found here: The transcriptome profiles and corresponding clinical data of LADC patients were downloaded from TCGA (https://portal.gdc.cancer.gov/) and GEO (https://www.ncbi.nlm.nih.gov/geo/) databases.

## Author Contributions

JC and YL involved in study concept and design. JC and YL involved in acquisition of data. CZ and YL involved in analysis and interpretation of data. JC and CZ drafted the manuscript. YL and JC involved in critical revision of the manuscript for intellectual content. All authors contributed to the article and approved the submitted version.

## Funding

This study was supported by The Project of Jiangxi Education Department (No. GJJ200223 and No. GJJ170122).

## Conflict of Interest

The authors declare that the research was conducted in the absence of any commercial or financial relationships that could be construed as a potential conflict of interest.

## Publisher’s Note

All claims expressed in this article are solely those of the authors and do not necessarily represent those of their affiliated organizations, or those of the publisher, the editors and the reviewers. Any product that may be evaluated in this article, or claim that may be made by its manufacturer, is not guaranteed or endorsed by the publisher.
