# Supplementary figures and images for: Establishing a Macrophage Phenotypic Switch-Associated Signature-Based Risk Model for Predicting the Prognoses of Lung Adenocarcinoma
Source: Front Oncol. 2022 Feb 23;11:771988. doi: 10.3389/fonc.2021.771988 (PMC8905507; doi:10.3389/fonc.2021.771988)

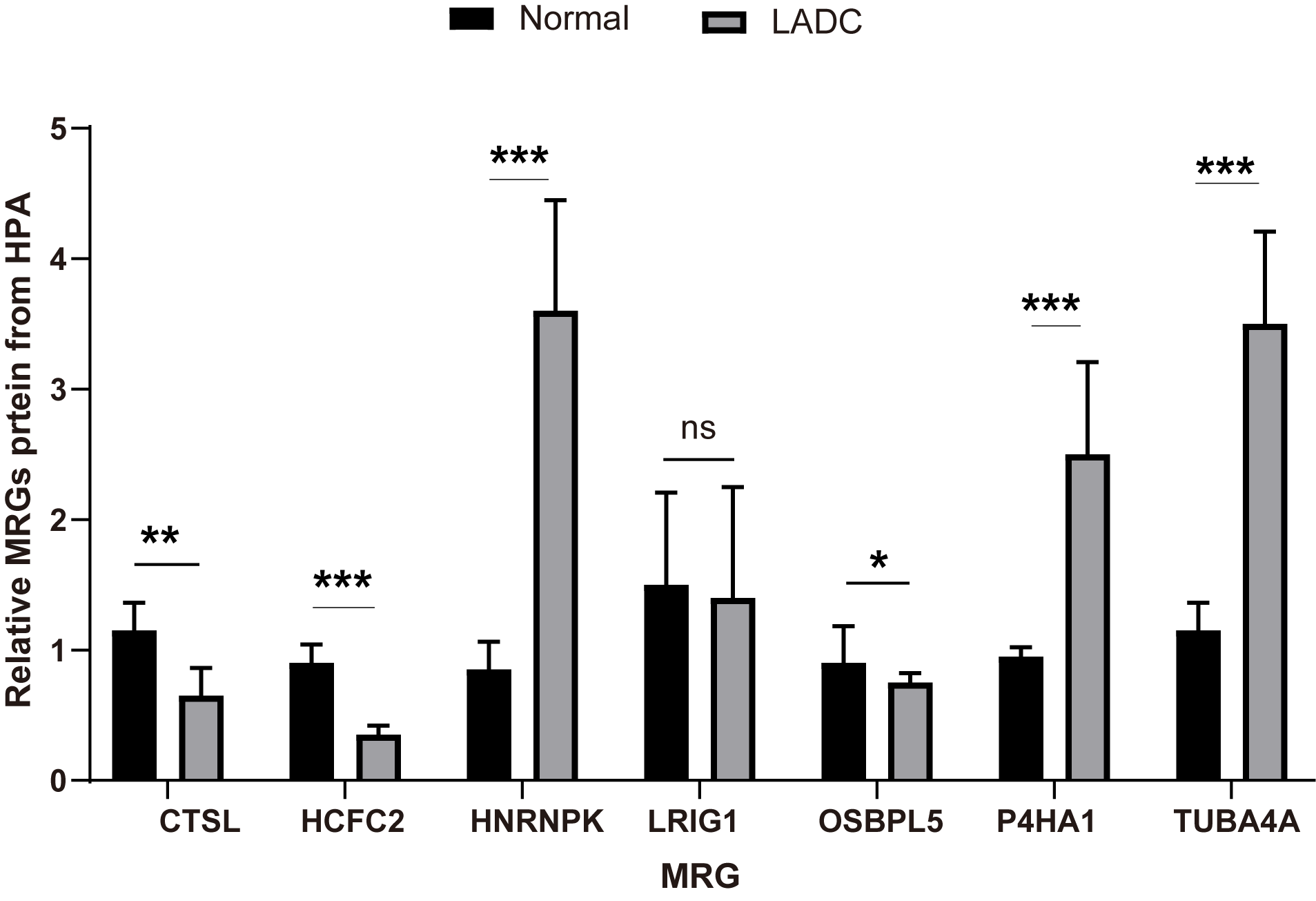

Supplement: Supplementary file 1 [file Image_1.tif]

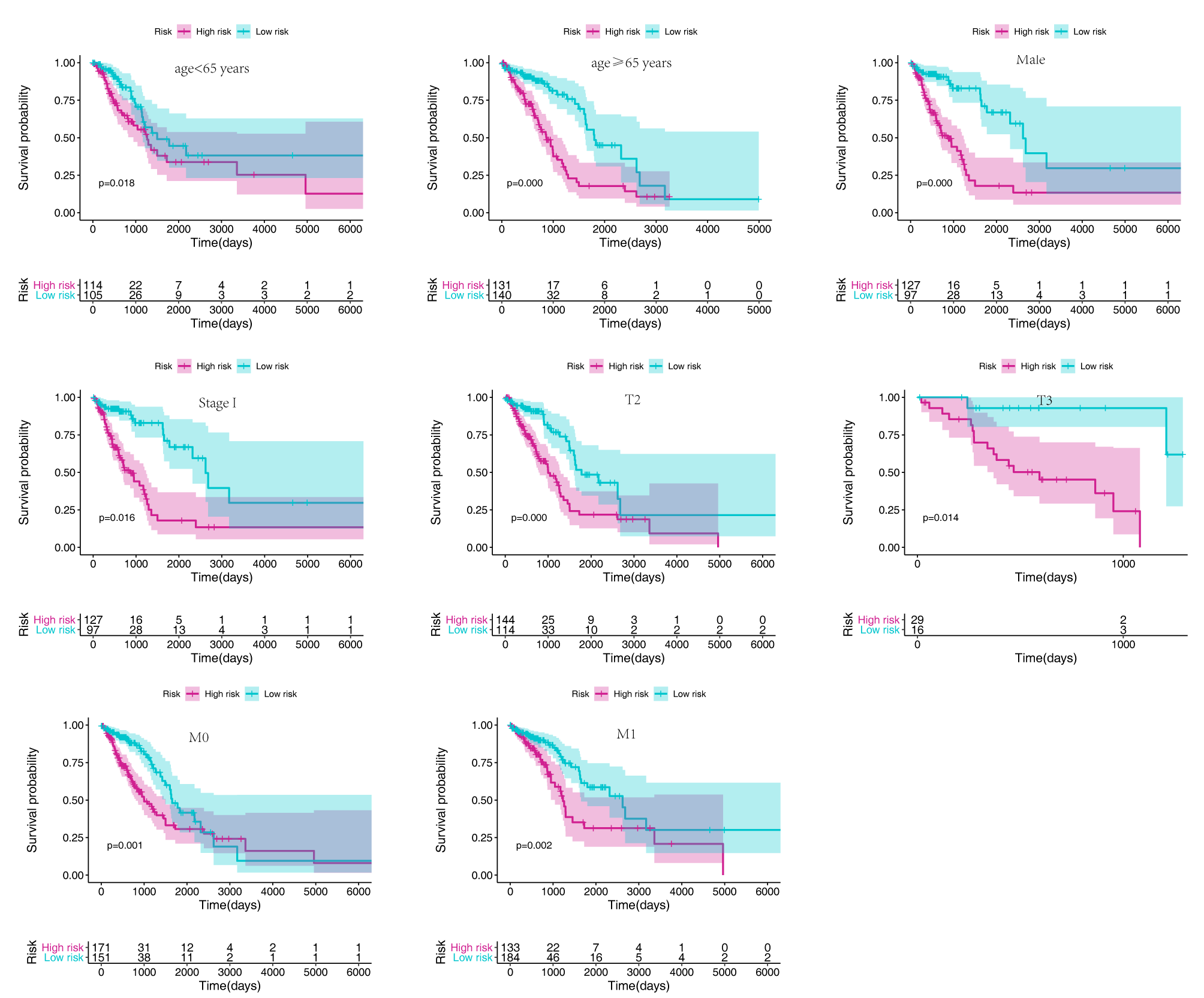

Supplement: Supplementary file 2 [file Image_2.tif]

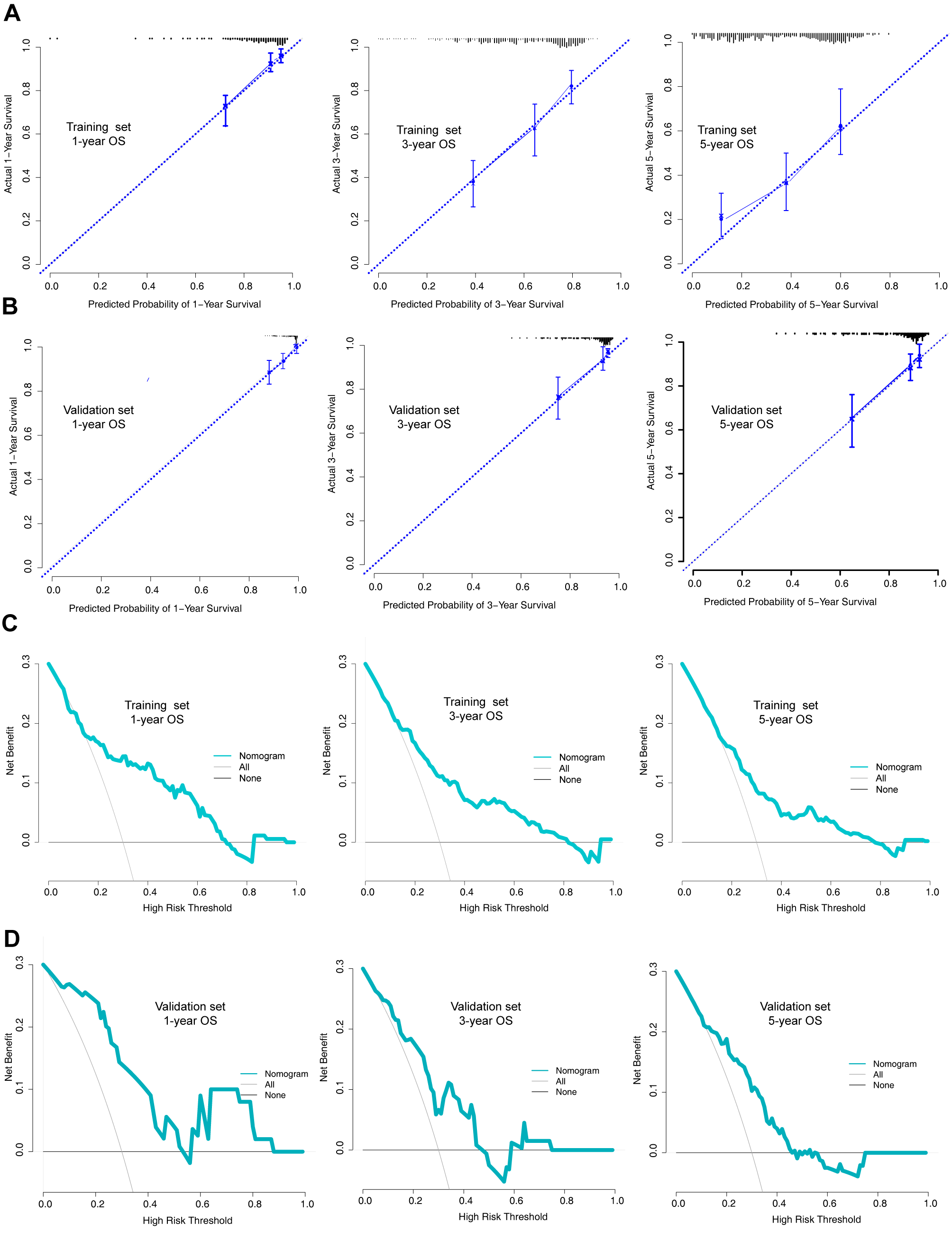

Supplement: Supplementary file 3 [file Image_3.tif]
