# Supplementary material for: Establishing a Macrophage Phenotypic Switch-Associated Signature-Based Risk Model for Predicting the Prognoses of Lung Adenocarcinoma
Source: Front Oncol. 2022 Feb 23;11:771988. doi: 10.3389/fonc.2021.771988 (PMC8905507; doi:10.3389/fonc.2021.771988)
Supplement: Supplementary file 5 [file Table_2.docx]

| **Table S2.** Prognostic roles of the MRGs signature with different demographic and clinical characteristics in GSE31210 validation set. | | | | | | | |
| --- | --- | --- | --- | --- | --- | --- | --- |
| **Characteristics** | **No.** | | **%** | **OS** | | **RFS** | |
|  | **high-risk** | **high-risk** |  | **HR (95% CI)** | ***P* value** | **HR (95% CI)** | ***P* value** |
| **Age (years)** |  |  | | | | | |
| < 65 | 87 | 77 | 72.57% | 0.357(0.131-0.976) | 0.045 | 0.611(0.325-1.150) | 0.127 |
| ≥ 65 | 26 | 36 | 27.43% | 0.363(0.122-1.085) | 0.070 | 0.407(0.174-0.952) | 0.038 |
| **Sex** |  |  | | | | | |
| Male | 60 | 45 | 46.46% | 0.343(0.114-1.033) | 0.343 | 0.399(0.179-0.889) | 0.025 |
| Female | 53 | 68 | 53.54% | 0.482(0.175-1.328) | 0.158 | 0.757(0.378-1.514) | 0.431 |
| **Smoking status** |  |  | | | | | |
| Ever smoker | 64 | 47 | 49.12% | 0.342(0.114-1.024) | 0.055 | 0.371(0.168-0.821) | 0.014 |
| Never smoker | 49 | 66 | 50.88% | 1.032(0.497-2.142) | 0.933 | 0.846(0.413-1.735) | 0.649 |
| **Stage** |  |  | | | | | |
| I | 73 | 95 | 74.34% | 0.174(0.050-0.606) | 0.006 | 0.506(0.262-0.976) | 0.042 |
| II | 40 | 18 | 25.66% | 1.498(0.579-3.875) | 0.405 | 1.240(0.555-2.768) | 0.600 |
| **Mutation** |  |  | | | | | |
| *ALK* fusion | *7* | 4 | 4.87% | 174.444(0.00-74410454) | 0.435 | 274.488(0.000-455660510) | 0.442 |
| *EGFR* mutation | *56* | 71 | 56.19% | 0.328(0.103-1.047) | 0.060 | 0.573(0.278-1.179) | 0.130 |
| *KRAS* mutation | *14* | 6 | 8.85% | 0.029(0.000-1315.453) | 0.518 | 0.432(0.050-3.707) | 0.444 |
| Wild-type *EGFR/**KRAS/ALK* | 36 | 32 | 30.09% | 0.339(0.109-1.058) | 0.062 | 0.404(0.175-0.930) | 0.033 |
| NA, Not available | | | | | | | |
